# Molecular testing to deliver personalized chemotherapy recommendations: risking over and undertreatment

**DOI:** 10.1186/s12916-022-02589-6

**Published:** 2022-11-09

**Authors:** Timothée Olivier, Vinay Prasad

**Affiliations:** 1 Department of Oncology, Geneva University Hospital, 4 Gabrielle-Perret-Gentil Street, 1205 Geneva, Switzerland; 2 Department of Epidemiology and Biostatistics, University of California San Francisco, 550 16th St., 2nd Fl, San Francisco, CA 94158 USA

**Keywords:** Adjuvant therapy, Oncology, Molecular test, Gene signature, ctDNA, Evidence-based medicine, Non-inferiority

## Abstract

**Background:**

In the adjuvant setting of cancer treatment, de-escalation strategies have the goal of omitting or minimizing treatment in patients, without compromising outcomes. Historically, eligibility for adjuvant treatment solely relied on the patient’s clinical and tumor’s pathological characteristics. At the turn of the century, based on new biological understanding, molecular-based strategies were tested and sometimes implemented.

**Main body:**

However, we illustrate how molecularly based de-escalation strategies may paradoxically lead to overtreatment. This may happen when the novel approach is tested in lieu of standard management and may not yield the same results when being implemented in addition to usual practice. In the DYNAMIC trial, adjuvant chemotherapy decision in stage II colon cancer was compared between a circulating tumor DNA (ctDNA)-based approach and the standard care. We show this may result in more patients receiving oxaliplatin-based chemotherapy and may expose a similar proportion of patients to chemotherapy if the novel strategy is implemented in addition to usual practice. The other potential risk is undertreatment. We provide an illustration of early breast cancer, where the decision of adjuvant chemotherapy based on the gene expression signature MammaPrint may lead to inferior outcomes as compared with the clinico-pathologic strategy. This may also happen when non-inferiority designs have large margins. Among solutions, it should be acknowledged that clinico-pathological features, like T4 in colon cancer, may not be abandoned and replaced by novel strategies in real-life practice. Therefore, novel strategies should be tested in addition to standard of care, and not *in lieu of*. Second, de-escalation trials should focus on the settings where the standard of care has a widespread agreement. This would avoid the risk of testing non-inferiority against an ineffective therapy, which guarantees successes without providing informative data.

**Conclusion:**

Simply because a molecular test is rational does not mean it can improve patient outcomes. Here, we highlight how molecular test-based strategies may result in either overtreatment or undertreatment. In the rapidly evolving field of medicine, where technological advances may be transformative, our piece highlights scientific pitfalls to be aware of when considering running such trials or before implementing novel strategies in daily practice.

## Background

De-escalation strategies have the desirable goal of omitting or minimizing treatment in patients, without compromising long-term outcomes [1]. While novel molecularly based de-escalation strategies promise to achieve this objective, at times, they may paradoxically increase the cumulative amount of treatment received by patients. Large non-inferior margins may also miss the loss of efficacy. We illustrate these questions and propose solutions to overcome the unintended consequences of molecularly based de-escalation strategies.

### Expected benefits from de-escalation strategies

In the adjuvant setting, patients have typically undergone curative surgery, and no tumor is visible on conventional radiography. The aim, in this setting, is to increase the chances of cure by eradicating microscopic disease with the goal of avoiding relapse [2, 3]. However, “increasing chances of cure” is only relevant at the group level, because a fraction of patients will be cured by the adjuvant therapy, while others would not derive any benefit. Moreover, to date, in solid tumor adjuvant settings, those who benefit are always a minority of treated patients [3].

Therefore, identifying who benefits from adjuvant treatment and who does not may spare some patients unnecessary treatment. Omitting toxic treatments offers direct physical and psychological benefits, avoiding the risk of short-term and long-term toxicities and quality of life impairment [4]. The cost of anti-cancer drugs continues to rise [5], with financial burden identified as a specific toxicity for patients with cancer. Being diagnosed with cancer confers a higher risk for bankruptcy [6], and a retrospective study suggested bankruptcy was associated with a higher risk of mortality after cancer diagnosis [7]. A systematic review found that half of the patients suffer from financial burden, and this was affecting the quality of life and treatment adherence [8]. In the adjuvant setting, drugs approved by the US Food and Drug Administration (FDA) between 2018 and 2022 carried a median cost of $158,000 per patient for a complete treatment, with a median cost to avert one event of $1,610,000, a cost that may not be sustainable for societies [9].

### Different tools to guide adjuvant decision

Historically, the identification of patients eligible for adjuvant treatment solely relied on the patient’s clinical and tumor’s pathological characteristics [3]. Specific tumor biologic features, gradually integrated into the pathological assessment over time, such as hormone receptor status [10] or human epidermal growth factor receptor 2 (HER2) expression for patients with breast cancer [11], and microsatellite instability (MSI) assessment in colorectal cancer [12, 13], also became guiding factors for adjuvant treatment decisions. At the turn of the century, based on new molecular understanding and techniques, gene expression signatures held the promise to refine adjuvant decisions, initially in breast cancer [14]. Some of these signatures (e.g., Oncotype DX or MammaPrint) were later prospectively studied and are used in clinical practice [15]. Circulating tumor cells (CTCs) or circulating tumor DNA (ctDNA) are also studied as potential biomarkers to guide adjuvant treatment [16].

### De-escalation strategies resulting in their opposite effect: more treatment

Theoretical advantages of new molecular tools rely on their ability to improve patient selection upon existing practice. Firstly, de-escalation strategies may result in opposite effects (more treatment), if the new approach is tested in lieu of the current practice, and not in addition to, which is not the way physicians will implement new strategies. An example of this occurred in the Circulating Tumour DNA Analysis Informing Adjuvant Chemotherapy in Stage II Colon Cancer (DYNAMIC) trial, which randomly assigned a 2 to 1 ratio of patients with stage II colon cancer between a ctDNA-guided approach and a standard approach to inform adjuvant treatment decision [17]. This was a non-inferiority trial designed to demonstrate that a ctDNA-guided approach would reduce the use of adjuvant treatment with no detrimental effect on the risk of recurrence. The trial met its primary endpoint, being non-inferiority in recurrence-free survival at 2 years, 93.5% in the ctDNA group and 92.4% in the standard management group, respectively, with a lower proportion of patients receiving adjuvant chemotherapy in the ctDNA-guided group as compared with standard management (15% versus 28%; relative risk = 1.82; 95% confidence interval, 1.25 to 2.65) [17].

Among the clinicopathological criteria defining high-risk patients with stage II colon cancer is T4 tumor and is included in international practice guidelines such as the European Society of Medical Oncology (ESMO) or the American Society of Clinical Oncology (ASCO) guidelines [18, 19]. A large matched-pair analysis of 3986 patients with T4 Union for International Cancer Control (UICC) stage II colon cancer showed 5-year survival rates of 70.9% with adjuvant treatment compared to 59.8% with no further therapy (*p* < 0.001) [20]. In the DYNAMIC trial, 32 out of 44 patients (72.7%) with T4 tumor did not receive adjuvant chemotherapy based on the ctDNA result, as compared with 30% (6 out of 20) of patients with T4 lesion who did not undergo further therapy in the standard management arm.

In the DYNAMIC trial, within the ctDNA-guided patients, those with a ctDNA negative result (thus not receiving chemotherapy) and T4 tumors presented inferior outcomes in terms of disease-free survival as compared with the ctDNA positive (thus receiving chemotherapy): 81.3% versus 86.4% were alive and disease-free after 3 years, respectively [17]. This finding led to the following comment, on social media, by Dr. Jeremy Jones: “My take home points from DYNAMIC: for truly high-risk patients (T4) do not skip chemo even if ctDNA (-). For low risk with (+) ctDNA seems reasonable to offer chemo. Bottom line if positive I trust the test; if negative I’m still suspicious.” [21].

As a result, it is likely in clinical practice that physicians will continue to consider this pathologic feature to inform their decision. If doctors adopted this strategy (treating all T4 patients in the ctDNA-guided group, regardless of the result of the test)—which we believe is entirely possible in real-life settings—then 26% of patients would receive adjuvant therapy in the ctDNA group, as compared with 28% in the standard arm. As a first consequence, the primary goal of the ctDNA approach, sparing chemotherapy, would evaporate. By only considering a single high-risk clinico-pathological feature—T4—among many others, this example demonstrates how the novel approach, being tested in lieu of standard management, may not yield the same results when being implemented in addition to usual practice. Clinical trials should focus on the latter question to preserve relevance to the real world.

Secondly, in the DYNAMIC trial, a higher proportion of patients undergoing adjuvant treatment received doublet oxaliplatin-based chemotherapy in the ctDNA-guided group (62%) compared with patients where standard management guided the decision (10%). When considering all patients enrolled in the study, and despite the fact that fewer patients received chemotherapy in the ctDNA group, a higher proportion of patients received oxaliplatin in the ctDNA group (9.5% vs 2.7% with standard management).

In other words, a patient with stage II colon cancer undergoing the ctDNA test will face a higher chance (or risk) of receiving oxaliplatin-doublet chemotherapy than a patient treated the conventional way. However, avoiding long-term oxaliplatin-induced neurotoxicity is a constant challenge in adjuvant shared decision of colon cancer adjuvant treatment [22]. Indeed, long-term oxaliplatin-induced neuropathy was present in two-thirds of patients after digestive adjuvant treatment, impairing their quality of life [23, 24].

The authors of the DYNAMIC trial contend that this result (higher rates of oxaliplatin-based chemotherapy in the ctDNA group) was mostly explained by the “known prognostic significance” of positive ctDNA results [17]. However, ctDNA positivity was not considered a high-risk feature before the study was run [19], and details for reasons supporting the choice of oxaliplatin-based chemotherapy were not reported.

There is an ambiguity in the DYNAMIC trial, where on the one hand, the first step of the decision, i.e., the choice of undergoing adjuvant chemotherapy or not, was decided exclusively according to the ctDNA result, but on the other hand, the second step of the decision (i.e., the type of chemotherapy and whether oxaliplatin should be added) was left “at the clinician’s discretion.” This demonstrates that the DYNAMIC trial, per protocol, was not exploring a “ctDNA exclusive” strategy, because an important part of the decision-making process was still relying on traditional clinico-pathological features. This may further explain why it is likely that oncologists will add the ctDNA results without dismissing their current risk assessment, not only when deciding the type of regimen, but also at the very first step of deciding whether a patient should be proposed adjuvant chemotherapy or not.

Therefore, it is entirely possible that contrary to the reported result of the DYNAMIC trial, the addition of a ctDNA-based approach to current practice would not result in fewer patients receiving adjuvant chemotherapy, which was the primary goal of the DYNAMIC trial, as doctors will engage in compensatory behavior. Also, it would lead to more patients receiving oxaliplatin-based doublets, known for specific long-term toxicity.

### De-escalation strategies in settings where current practice has questionable benefit

Another complexity with interpreting molecular tests that permit us to exclude therapy is that the fundamental efficacy of those therapies may have shifted or eroded over time. Stage II colon cancer is one such place [25]. Among trials supporting the benefit of adjuvant treatment in stage II colon cancer, most took place decades ago and no longer reflect modern practice. These trials were considered by the ASCO guideline expert panel to be “low” or “very-low” quality evidence [19]. The implementation of modern surgery techniques has shown improvement in outcomes in many retrospective series [26–29]. Stage migration due to better imaging and optimization of surgical staging [30] may also have played a role in a trend toward better outcomes over time [25]. As an illustration, the 2-year recurrence-free survival in the DYNAMIC trial was around 92-93% in both arms, when we estimated that no more than 88.4% of patients were free of recurrence and alive after 2 years in the QUASAR trial (older) comparing adjuvant chemotherapy to observation in stage II cancer [31].

These changes have relevance for the molecular test in DYNAMIC. If the gains of stage II treatment have gotten smaller over the years, a test, even an inaccurate or ineffective one, can easily show noninferiority over the current standard of care—precisely because the gains of the current strategy are smaller.

Consider a thought experiment: let us assume no benefit from the current clinico-pathological guided adjuvant strategy in stage II colon cancer. Now, imagine one proposes an innovative test-based strategy, as in the DYNAMIC trial, to replace the old algorithm. If your new strategy demonstrates to result in the same outcomes: is it “as good” or “as useless”? Even randomly omitting chemotherapy would result in the same survival outcomes. In other words, proving non-inferiority against a strategy which is itself subject to equipoise and not strongly supported is questionable and may result in a guarantee that your trial will yield similar outcomes.

### Loss of efficacy: a potential downside in de-escalation strategies

Consider now early breast cancer. In patients with a high-risk clinical feature, such as lymph node involvement, the National Comprehensive Cancer Network (NCCN) recommends that the decision to undergo adjuvant treatment can be guided by the 70-gene signature MammaPrint result. This is based on the MINDACT study, which prospectively compared patients with high clinical risk and low genomic risk (per MammaPrint), after being randomized between chemotherapy and not [32]. The goal of the MINDACT study was to test whether omitting cytotoxic treatment in this subgroup could be done without impairing efficacy.

The study was positive, with 94.7% of patients with high clinical risk and low genomic risk, and not receiving chemotherapy, being alive without metastases after 5 years. The lower boundary of the 95% confidence interval (92.5 to 96.2) was higher than 92%, which was defined as the threshold for the primary endpoint of the trial.

However, when compared with patients of the same group receiving chemotherapy, the rate of 5-year survival without metastasis was, on average, 1.5 percentage points lower [32]. Also, these patients, when receiving chemotherapy based on the clinical risk assessment, had a higher 93.3% 5-year disease-free-survival (DFS) compared with 90.3% in patients not receiving chemotherapy based on MammaPrint result (hazard ratio = 0.64; *p* = 0.03). What tradeoffs are clinically acceptable for de-escalation strategies [33]? The primary endpoint of the MINDACT trial did not directly answer this question.

Non-inferiority trials are often used to test de-escalation [34]. However, a lack of transparency in how the margin is chosen may limit the trial definite conclusion [35]. In other words, when margins are too large, a positive non-inferiority trial result may translate into a clinical detriment. In the DYNAMIC trial, testing the ctDNA approach in stage II colon cancer, the non-inferiority margin was 8.5 percentage points for the analysis of 2-year recurrence-free survival. This margin is large. It is higher than the observed benefit, in terms of recurrence rate, from adjuvant treatment versus observation in stage II colon cancer in the QUASAR trial (4.8 percentage points difference) [31]. Large non-inferiority margins may guarantee the success of such trials without ensuring similar efficacy for patients.

### Possible solutions

Solutions to our dilemma of molecular testing should balance a desire to use and embrace new technology with the need to appraise it rigorously in well-done studies. As the first step, novel test-based de-escalation strategy trial should more closely mimic the ultimate clinical practice and ask if the new strategy is better in addition to conventional care and not “in lieu of” it.

A second strategy would be to limit de-escalation trials to settings where the current practice benefits have reach a widespread agreement. This would avoid the risk of testing non-inferiority against an ineffective therapy, which guarantees successes but provide no informative data. In the case of DYNAMIC, stage III colon cancer is a better target. If, however, a trial wish to be run in settings where equipoise remains, adding a third arm with even less treatment administration—perhaps done at random or based on clinicopathologic information—would allow to better assess the true effect of the novel strategy.

The third solution tackles the issue raised by non-inferiority margins. Test-based decision strategies should not be limited to de-escalation trials and be superiority trials instead. For instance, in stage III colon cancer, among those not receiving chemotherapy based on current management, does ctDNA detect a subgroup that may benefit from adjuvant chemotherapy?

## Conclusions

Simply because a molecular test is rational does not mean it can improve patient outcomes. Among patients with ovarian cancer, closely following patients with the tumor marker cancer antigen 125 (CA-125), and initiating therapy upon its rise, famously did not confer a survival benefit [36]. The same is true for efforts to tailor treatment, or de-escalate. These do not guarantee patient benefits. Here, we highlight, based on 2 examples, how molecular test-based strategies may result in either overtreatment (ctDNA approach in stage II colon cancer) or undertreatment (gene signature-based adjuvant chemotherapy in early breast cancer). De-escalation trials face a tension. Is their primary goal to advance the market share of specific products, or is it to provide clarity regarding which patients truly benefit from therapy? If it is the latter, clearly, study design is needed to ensure the trial informs clinical practice. In the rapidly evolving field of medicine, where technological advances may be transformative, our piece highlights scientific pitfalls to be aware of when considering running such trials or before implementing novel strategies.

## Data Availability

All data on which this work was based are publicly available.

## Abbreviations

ctDNA: Circulating tumor DNA
FDA: US Food and Drug Administration
HER2: Human epidermal growth factor receptor 2
MSI: Microsatellite instability
CTCs: Circulating tumor cells
ESMO: European Society of Medical Oncology
ASCO: American Society of Clinical Oncology
UICC: Union for International Cancer Control
NCCN: National Comprehensive Cancer Network
DFS: Disease-free-survival
CA-125: Cancer antigen 125
